# [μ-3,3′-Diisopropyl-1,1′-(propane-1,3-di­yl)bis­(1,3-diazinan-2-yl­idene)]bis­[bromido­(η^4^-cyclo­octa-1,5-diene)rhodium(I)]

**DOI:** 10.1107/S1600536814001135

**Published:** 2014-01-31

**Authors:** Gajanan Manohar Pawar, Klaus Wurst, Dongren Wang, Michael Buchmeiser

**Affiliations:** a Institut für Polymerchemie, Universität Stuttgart, Pfaffenwaldring 55, D-70569 Stuttgart, Germany; b Institut für Allgemeine Anorganische und Theoretische Chemie, Universität Innsbruck, Innrain 80/82, 6020 Innsbruck, Austria

## Abstract

The title compound, [Rh_2_Br_2_(C_8_H_12_)_2_(C_17_H_32_N_4_)], was obtained by the reaction of 3,3′-(propane-1,3-di­yl)bis­(1-isopropyl-3,4,5,6-tetra­hydro­pyrimidin-1-ium) bromide and [{Rh(cod)Cl}_2_] (cod is cyclo­octa-1,5-diene) in tetra­hydro­furan. The two Rh^I^ atoms each have a distorted square-planar coordination environment, defined by a bidentate cod ligand, a bromide anion and one C atom of the bridging bidentate bis-*N*-heterocyclcic carbene (NHC) ligand. The average Rh—C_NHC_ distance is 2.038 (7) Å, suggesting that the bond has a major σ contribution with very little back donation. The distances between the cod ligands and the Rh^I^ atoms vary between 2.104 (4) and 2.210 (4) Å.

## Related literature   

For general background on the development of *N*-heterocyclic carbenes (NHC) as replacements for phosphines in the area of organometallic catalysis and Rh–NHC-based complexes, see: Herrmann *et al.* (1996 ▶, 1997 ▶); Mayr *et al.* (2004 ▶); Díez-González *et al.* (2009 ▶). For examples of the application of Rh^I^ complexes as catalysts in hydro­formyl­ation reactions, see: Evans *et al.* (1968 ▶); Reindl *et al.* (2013 ▶). For the synthesis of homobimetallic Rh^I^–NHC complexes and their application as catalysts in hydro­silylations, see: Huckaba *et al.* (2013 ▶).
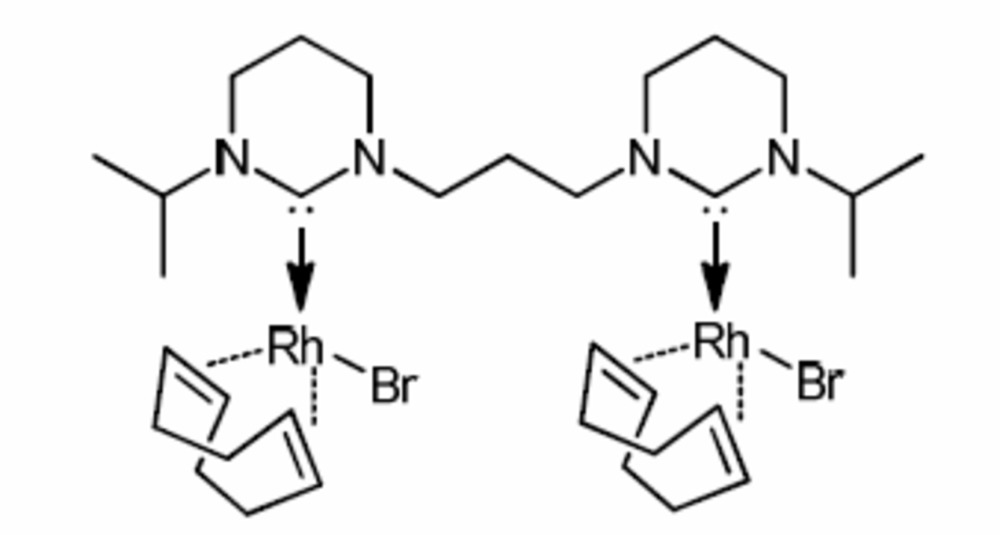


## Experimental   

### 

#### Crystal data  

[Rh_2_Br_2_(C_8_H_12_)_2_(C_17_H_32_N_4_)]
*M* *_r_* = 874.46
Orthorhombic, 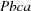

*a* = 16.4075 (2) Å
*b* = 15.7975 (3) Å
*c* = 27.1065 (4) Å
*V* = 7025.94 (19) Å^3^
*Z* = 8
Mo *K*α radiation
μ = 3.24 mm^−1^
*T* = 233 K
0.20 × 0.10 × 0.08 mm

#### Data collection  

Nonius KappaCCD diffractometer
Absorption correction: multi-scan (*SORTAV*; Blessing, 1995 ▶) *T*_min_ = 0.432, *T*_max_ = 0.755
57878 measured reflections
6186 independent reflections
5045 reflections with *I* > 2σ(*I*)
*R*_int_ = 0.083

#### Refinement  

*R*[*F* ^2^ > 2σ(*F* ^2^)] = 0.033
*wR*(*F* ^2^) = 0.078
*S* = 1.06
6186 reflections
402 parameters
8 restraints
H atoms treated by a mixture of independent and constrained refinement
Δρ_max_ = 0.95 e Å^−3^
Δρ_min_ = −0.51 e Å^−3^

### 

Data collection: *COLLECT* (Nonius, 1998 ▶); cell refinement: *DENZO-SMN* (Otwinowski & Minor, 1997 ▶); data reduction: *DENZO*/*SCALEPACK* (Otwinowski & Minor, 1997 ▶); program(s) used to solve structure: *SHELXS86* (Sheldrick, 2008 ▶); program(s) used to refine structure: *SHELXL97* (Sheldrick, 2008 ▶); molecular graphics: *SHELXTL* (Sheldrick, 2008 ▶); software used to prepare material for publication: *SHELXTL* and *publCIF* (Westrip, 2010 ▶).

## Supplementary Material

Crystal structure: contains datablock(s) I, New_Global_Publ_Block. DOI: 10.1107/S1600536814001135/im2446sup1.cif

Structure factors: contains datablock(s) I. DOI: 10.1107/S1600536814001135/im2446Isup2.hkl

CCDC reference:

Additional supporting information:  crystallographic information; 3D view; checkCIF report

## Figures and Tables

**Fig. 1.**
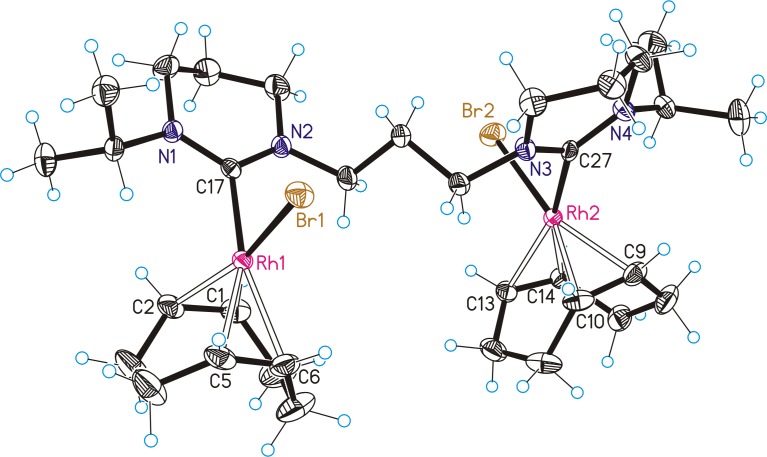
Molecular structure of the title molecule with displacement ellipsoids drawn at the 30% probability level.

## supplementary crystallographic information

### 1. Comment

The hydroformylation reaction is one of the most important catalytic reactions at an industrial level that can be employed for the conversion of alkenes, carbon monoxide and hydrogen into aldehydes and alcohols. Most of the hydroformylation catalysts so far were based on rhodium and phosphine ligands (*e.g.*, the Wilkinson-catalyst [RhCl(PPh_3_)_3_] (Evans *et al.*, 1968). However, phosphine ligands have some common disadvantages as they are easily oxidized by molecular oxygen in solution. Furthermore, phosphines and CO show similar binding constants to rhodium. Due to the fact that CO-pressures applied during hydroformylation are quite high, an excess of phosphine is required to generate a sterically demanding environment around the active rhodium-center, a prerequisite for high n/iso-ratios. N-heterocyclic carbenes (NHCs), owing to their strong σ donation and the excellent stability, have been widely applied as an ideal replacement for phosphines in the area of organometallic catalysis (Díez-González *et al.*, 2009). Within that context, homobimetallic rhodium(I) NHC complexes have been reported to represent versatile catalysts for the hydrosilylation of *e.g.* aldehydes, ketones, alkenes, nitriles, isocyanates and tertiary amides. (Huckaba *et al.*, 2013). The title compound was prepared by the reaction of 3,3'- (propane-1,3-diyl)bis(1-isopropyl-3,4,5,6-tetrahydropyrimidin-1-ium) bromide and [{Rh(cod)Cl}_2_] in anhydrous THF. It crystallizes in the space group Pbca (No.61). The structure exhibits a typical pesudo-square planar ligand environment for the Rh(I) centers which are coordinated by a bidentate cycloocta-1,5-diene (cod) ligand, one carbon atom of the bridging bis-N-heterocyclic carbene ligand and one bromide atom. The molecular structure is closely similar to that of the recently reported compounds bromo(η^4^-1,5-cyclooctadiene){1,3-bis(2-propyl)-3,4,5,6- tetrahydropyrimidin- 2-ylidene}rhodium and bromo(η^4^-1,5-cyclooctadiene){1,3-dimesityl-3,4,5,6- tetrahydropyrimidin- 2-ylidene}rhodium (Mayr *et al.*, 2004).

### 2. Experimental

[{Rh(cod)Cl}_2_] (200 mg, 0.47 mmol) was dissolved in anhydrous THF (5 ml), and lithium *tert*-butoxide (91 mg, 1.14 mmol) was added under vigorous stirring. The mixture was stirred for another 30 min at room temperature, then 3,3'-(propane-1,3-diyl)bis(1-isopropyl-3,4,5,6-tetrahydropyrimidin-1-ium)bromide (220 mg, 0.49 mmol) was added. The reaction mixture was stirred overnight at 65°C, after this time TLC showed no further conversion. The solvent was removed in *vacuo* and the product was purified by column chromatography (silica gel) using dichloromethane:ethanol (250:4) as the mobile phase. The product eluted as a yellow band in the second fraction. The product fractions were pooled and evaporated to dryness to yield a yellow solid (230 mg, 56%). Yellow crystals suitable for X-ray analysis were obtained by layering pentane over a dilute solution of the title compound in CH_2_Cl_2_ at -30°C.

### 3. Refinement

All hydrogen positions could be localised, but were only refined regularly with bond restraints of 93 pm at the double bonds of cyclooctadiene (C1, C2, C5, C6, C9, C10, C13 and C14). All other hydrogens were calculated by geometrical methods and refined as a riding model with temperature factors U_eq_ 1.2 or 1.5 (methyl groups) times higher than their linked carbon atoms.

### Crystal data

**Table:**

[Rh_2_Br_2_(C_8_H_12_)_2_(C_17_H_32_N_4_)]	*F*(000) = 3536
*M**_r_* = 874.46	*D*_x_ = 1.653 Mg m^−^^3^
Orthorhombic, *P**b**c**a*	Mo *K*α radiation, λ = 0.71073 Å
Hall symbol: -P 2ac 2ab	Cell parameters from 54150 reflections
*a* = 16.4075 (2) Å	θ = 1.0–25.0°
*b* = 15.7975 (3) Å	µ = 3.24 mm^−^^1^
*c* = 27.1065 (4) Å	*T* = 233 K
*V* = 7025.94 (19) Å^3^	Prism, yellow
*Z* = 8	0.2 × 0.1 × 0.08 mm

### Data collection

**Table:**

Nonius KappaCCD diffractometer	6186 independent reflections
Radiation source: fine-focus sealed tube	5045 reflections with *I* > 2σ(*I*)
Graphite monochromator	*R*_int_ = 0.083
Detector resolution: 9.1 pixels mm^-1^	θ_max_ = 25.0°, θ_min_ = 1.9°
phi and ω scans	*h* = −19→19
Absorption correction: multi-scan (*SORTAV*; Blessing, 1995)	*k* = −18→18
*T*_min_ = 0.432, *T*_max_ = 0.755	*l* = −32→32
57878 measured reflections	

### Refinement

**Table:**

Refinement on *F*^2^	Primary atom site location: structure-invariant direct methods
Least-squares matrix: full	Secondary atom site location: difference Fourier map
*R*[*F*^2^ > 2σ(*F*^2^)] = 0.033	Hydrogen site location: inferred from neighbouring sites
*wR*(*F*^2^) = 0.078	H atoms treated by a mixture of independent and constrained refinement
*S* = 1.06	*w* = 1/[σ^2^(*F*_o_^2^) + (0.0254*P*)^2^ + 11.9446*P*] where *P* = (*F*_o_^2^ + 2*F*_c_^2^)/3
6186 reflections	(Δ/σ)_max_ = 0.002
402 parameters	Δρ_max_ = 0.95 e Å^−^^3^
8 restraints	Δρ_min_ = −0.51 e Å^−^^3^

### Special details

**Table:**

Experimental. Absorption correction: multi-scan from symmetry-related measurements
Geometry. All e.s.d.'s (except the e.s.d. in the dihedral angle between two l.s. planes) are estimated using the full covariance matrix. The cell e.s.d.'s are taken into account individually in the estimation of e.s.d.'s in distances, angles and torsion angles; correlations between e.s.d.'s in cell parameters are only used when they are defined by crystal symmetry. An approximate (isotropic) treatment of cell e.s.d.'s is used for estimating e.s.d.'s involving l.s. planes.
Refinement. Hydrogen atoms at C=C bonds of COD were refined with bond restraints (d=0.93 angs.) for C1, C2, C5, C6, C9, C10, C13 and C14

### Fractional atomic coordinates and isotropic or equivalent isotropic displacement parameters (Å^2^)

**Table:**

	*x*	*y*	*z*	*U*_iso_*/*U*_eq_	
Rh1	0.311925 (16)	0.057582 (18)	0.871181 (10)	0.02707 (9)	
Rh2	0.322674 (16)	0.166937 (18)	0.624003 (10)	0.02634 (9)	
Br1	0.21591 (3)	0.18233 (3)	0.873742 (16)	0.04127 (12)	
Br2	0.23333 (3)	0.03852 (3)	0.613041 (18)	0.04847 (13)	
N1	0.17169 (18)	−0.06234 (19)	0.87524 (11)	0.0312 (7)	
N4	0.17595 (18)	0.2779 (2)	0.61382 (11)	0.0316 (7)	
N2	0.20658 (19)	−0.0214 (2)	0.79627 (11)	0.0340 (8)	
N3	0.20629 (18)	0.2398 (2)	0.69400 (11)	0.0319 (7)	
C1	0.3988 (3)	−0.0285 (3)	0.84394 (18)	0.0462 (11)	
H1	0.376 (2)	−0.064 (2)	0.8205 (12)	0.050 (13)*	
C2	0.3797 (3)	−0.0498 (3)	0.89246 (18)	0.0455 (11)	
H2	0.3421 (19)	−0.090 (2)	0.9001 (15)	0.041 (12)*	
C3	0.4344 (4)	−0.0301 (4)	0.9360 (2)	0.0796 (18)	
H3A	0.4201	−0.0690	0.9628	0.096*	
H3B	0.4907	−0.0426	0.9264	0.096*	
C4	0.4320 (4)	0.0559 (4)	0.9555 (2)	0.0792 (18)	
H4A	0.4876	0.0729	0.9641	0.095*	
H4B	0.4000	0.0556	0.9861	0.095*	
C5	0.3968 (3)	0.1214 (3)	0.92170 (19)	0.0485 (12)	
H5	0.361 (2)	0.158 (2)	0.9372 (15)	0.059 (15)*	
C6	0.4213 (3)	0.1385 (3)	0.8752 (2)	0.0508 (12)	
H6	0.401 (2)	0.1895 (15)	0.8629 (12)	0.025 (9)*	
C7	0.4924 (3)	0.0970 (4)	0.8492 (3)	0.0798 (18)	
H7A	0.5115	0.1353	0.8231	0.096*	
H7B	0.5370	0.0905	0.8729	0.096*	
C8	0.4758 (3)	0.0144 (4)	0.8270 (2)	0.0811 (18)	
H8A	0.5218	−0.0232	0.8342	0.097*	
H8B	0.4730	0.0214	0.7911	0.097*	
C9	0.3832 (3)	0.2798 (3)	0.60524 (17)	0.0420 (10)	
H9	0.3462 (19)	0.3251 (18)	0.6024 (14)	0.034 (11)*	
C10	0.4035 (2)	0.2582 (3)	0.65399 (18)	0.0454 (11)	
H10	0.376 (2)	0.283 (2)	0.6803 (11)	0.049 (13)*	
C11	0.4850 (3)	0.2212 (4)	0.66950 (19)	0.0618 (14)	
H11A	0.5275	0.2435	0.6477	0.074*	
H11B	0.4973	0.2401	0.7031	0.074*	
C12	0.4878 (3)	0.1255 (4)	0.66804 (18)	0.0569 (13)	
H12A	0.5438	0.1076	0.6608	0.068*	
H12B	0.4734	0.1035	0.7007	0.068*	
C13	0.4314 (2)	0.0870 (3)	0.63017 (16)	0.0395 (10)	
H13	0.409 (2)	0.0370 (18)	0.6407 (15)	0.053 (13)*	
C14	0.4256 (2)	0.1116 (3)	0.58242 (16)	0.0385 (10)	
H14	0.399 (2)	0.076 (2)	0.5604 (11)	0.035 (11)*	
C15	0.4765 (3)	0.1803 (3)	0.55893 (17)	0.0521 (12)	
H15A	0.4877	0.1650	0.5246	0.063*	
H15B	0.5289	0.1840	0.5762	0.063*	
C16	0.4352 (3)	0.2658 (3)	0.56028 (19)	0.0592 (13)	
H16A	0.4770	0.3100	0.5589	0.071*	
H16B	0.4010	0.2716	0.5308	0.071*	
C17	0.2195 (2)	−0.0158 (2)	0.84536 (13)	0.0268 (8)	
C18	0.1040 (3)	−0.1135 (3)	0.85686 (16)	0.0452 (11)	
H18A	0.0546	−0.0787	0.8550	0.054*	
H18B	0.0936	−0.1604	0.8796	0.054*	
C19	0.1239 (3)	−0.1476 (3)	0.80667 (17)	0.0488 (11)	
H19A	0.1702	−0.1867	0.8089	0.059*	
H19B	0.0770	−0.1788	0.7935	0.059*	
C20	0.1447 (3)	−0.0753 (3)	0.77315 (16)	0.0486 (11)	
H20A	0.1654	−0.0972	0.7417	0.058*	
H20B	0.0956	−0.0419	0.7664	0.058*	
C21	0.2480 (2)	0.0378 (3)	0.76332 (14)	0.0395 (10)	
H21A	0.2705	0.0063	0.7353	0.047*	
H21B	0.2937	0.0637	0.7810	0.047*	
C22	0.1927 (2)	0.1073 (3)	0.74396 (14)	0.0373 (9)	
H22A	0.1545	0.1251	0.7697	0.045*	
H22B	0.1613	0.0866	0.7157	0.045*	
C23	0.2462 (2)	0.1819 (3)	0.72838 (13)	0.0355 (9)	
H23A	0.2623	0.2136	0.7579	0.043*	
H23B	0.2959	0.1600	0.7130	0.043*	
C24	0.1405 (3)	0.2910 (3)	0.71502 (15)	0.0438 (10)	
H24A	0.1573	0.3133	0.7472	0.053*	
H24B	0.0919	0.2560	0.7198	0.053*	
C25	0.1214 (3)	0.3630 (3)	0.68061 (17)	0.0451 (11)	
H25A	0.0730	0.3935	0.6923	0.054*	
H25B	0.1672	0.4028	0.6797	0.054*	
C26	0.1061 (3)	0.3280 (3)	0.63002 (16)	0.0447 (11)	
H26A	0.0572	0.2924	0.6305	0.054*	
H26B	0.0968	0.3746	0.6068	0.054*	
C27	0.2226 (2)	0.2346 (2)	0.64542 (13)	0.0264 (8)	
C28	0.1766 (2)	−0.0514 (2)	0.92925 (13)	0.0331 (9)	
H28	0.2234	−0.0136	0.9360	0.040*	
C29	0.1865 (2)	0.2654 (3)	0.55998 (14)	0.0368 (9)	
H29	0.2368	0.2317	0.5554	0.044*	
C281	0.1927 (3)	−0.1345 (3)	0.95619 (17)	0.0528 (12)	
H28A	0.2419	−0.1603	0.9433	0.079*	
H28B	0.1470	−0.1725	0.9512	0.079*	
H28C	0.1993	−0.1235	0.9912	0.079*	
C282	0.1006 (3)	−0.0072 (3)	0.94842 (16)	0.0490 (11)	
H28D	0.0925	0.0452	0.9305	0.073*	
H28E	0.1072	0.0051	0.9833	0.073*	
H28F	0.0537	−0.0437	0.9438	0.073*	
C291	0.1980 (3)	0.3475 (3)	0.53222 (18)	0.0625 (14)	
H29A	0.2437	0.3783	0.5462	0.094*	
H29B	0.1490	0.3814	0.5349	0.094*	
H29C	0.2088	0.3353	0.4978	0.094*	
C292	0.1166 (3)	0.2136 (3)	0.53902 (17)	0.0563 (13)	
H29D	0.1112	0.1614	0.5576	0.084*	
H29E	0.1276	0.2006	0.5047	0.084*	
H29F	0.0664	0.2458	0.5414	0.084*	

### Atomic displacement parameters (Å^2^)

**Table:**

	*U*^11^	*U*^22^	*U*^33^	*U*^12^	*U*^13^	*U*^23^
Rh1	0.02475 (15)	0.02505 (17)	0.03142 (17)	−0.00038 (12)	−0.00464 (12)	0.00325 (12)
Rh2	0.02445 (15)	0.02526 (17)	0.02930 (16)	0.00086 (12)	0.00006 (11)	0.00182 (12)
Br1	0.0385 (2)	0.0333 (2)	0.0520 (3)	0.00655 (18)	−0.00447 (18)	−0.00114 (19)
Br2	0.0444 (3)	0.0330 (2)	0.0680 (3)	−0.00669 (19)	−0.0033 (2)	−0.0044 (2)
N1	0.0352 (17)	0.0279 (17)	0.0307 (17)	−0.0085 (14)	−0.0037 (13)	0.0025 (14)
N4	0.0290 (16)	0.0339 (18)	0.0320 (18)	0.0062 (14)	−0.0016 (13)	0.0006 (14)
N2	0.0377 (18)	0.0363 (19)	0.0280 (18)	−0.0069 (15)	−0.0054 (13)	0.0036 (14)
N3	0.0322 (17)	0.0363 (19)	0.0272 (18)	0.0076 (14)	0.0031 (13)	0.0045 (14)
C1	0.036 (2)	0.041 (3)	0.062 (3)	0.011 (2)	0.001 (2)	−0.009 (2)
C2	0.040 (2)	0.031 (2)	0.065 (3)	0.007 (2)	−0.017 (2)	0.008 (2)
C3	0.095 (4)	0.066 (4)	0.077 (4)	0.015 (3)	−0.051 (3)	0.008 (3)
C4	0.104 (5)	0.061 (4)	0.073 (4)	−0.003 (3)	−0.051 (3)	0.003 (3)
C5	0.047 (3)	0.038 (3)	0.061 (3)	−0.004 (2)	−0.025 (2)	−0.005 (2)
C6	0.035 (2)	0.037 (3)	0.081 (4)	−0.009 (2)	−0.007 (2)	0.008 (2)
C7	0.047 (3)	0.075 (4)	0.118 (5)	−0.017 (3)	0.024 (3)	0.000 (4)
C8	0.048 (3)	0.090 (5)	0.105 (5)	0.000 (3)	0.026 (3)	−0.009 (4)
C9	0.037 (2)	0.028 (2)	0.061 (3)	−0.0046 (19)	0.007 (2)	0.001 (2)
C10	0.031 (2)	0.051 (3)	0.054 (3)	−0.008 (2)	−0.001 (2)	−0.017 (2)
C11	0.035 (2)	0.085 (4)	0.066 (3)	−0.003 (3)	−0.013 (2)	−0.016 (3)
C12	0.034 (2)	0.084 (4)	0.052 (3)	0.012 (2)	−0.005 (2)	0.014 (3)
C13	0.027 (2)	0.042 (3)	0.049 (3)	0.0093 (18)	0.0050 (18)	0.011 (2)
C14	0.034 (2)	0.038 (2)	0.043 (3)	0.0072 (19)	0.0063 (18)	−0.004 (2)
C15	0.048 (3)	0.063 (3)	0.046 (3)	0.002 (2)	0.016 (2)	0.009 (2)
C16	0.057 (3)	0.052 (3)	0.069 (3)	−0.011 (3)	0.022 (2)	0.017 (3)
C17	0.0278 (19)	0.0232 (19)	0.029 (2)	0.0052 (16)	−0.0032 (15)	0.0003 (15)
C18	0.044 (2)	0.042 (3)	0.049 (3)	−0.017 (2)	−0.002 (2)	−0.002 (2)
C19	0.059 (3)	0.038 (3)	0.050 (3)	−0.011 (2)	−0.008 (2)	−0.008 (2)
C20	0.060 (3)	0.048 (3)	0.038 (2)	−0.010 (2)	−0.016 (2)	−0.003 (2)
C21	0.039 (2)	0.051 (3)	0.029 (2)	0.002 (2)	0.0016 (17)	0.0095 (19)
C22	0.034 (2)	0.047 (3)	0.031 (2)	−0.0024 (18)	−0.0012 (16)	0.0110 (19)
C23	0.034 (2)	0.045 (2)	0.027 (2)	0.0012 (18)	−0.0025 (16)	0.0057 (18)
C24	0.046 (2)	0.046 (3)	0.040 (2)	0.010 (2)	0.0085 (19)	−0.003 (2)
C25	0.045 (2)	0.035 (2)	0.054 (3)	0.013 (2)	0.009 (2)	−0.003 (2)
C26	0.039 (2)	0.043 (3)	0.052 (3)	0.017 (2)	−0.0019 (19)	0.002 (2)
C27	0.0277 (18)	0.0199 (18)	0.032 (2)	−0.0014 (15)	0.0031 (15)	0.0058 (15)
C28	0.040 (2)	0.031 (2)	0.028 (2)	−0.0028 (17)	0.0012 (16)	0.0057 (17)
C29	0.040 (2)	0.042 (2)	0.029 (2)	0.0047 (19)	−0.0057 (17)	0.0047 (18)
C281	0.070 (3)	0.044 (3)	0.045 (3)	0.008 (2)	0.005 (2)	0.015 (2)
C282	0.051 (3)	0.048 (3)	0.048 (3)	0.008 (2)	0.008 (2)	0.003 (2)
C291	0.095 (4)	0.051 (3)	0.041 (3)	0.004 (3)	−0.004 (2)	0.014 (2)
C292	0.054 (3)	0.064 (3)	0.050 (3)	−0.006 (2)	−0.017 (2)	−0.002 (2)

### Geometric parameters (Å, º)

**Table:**

Rh1—C17	2.033 (4)	C12—H12A	0.9800
Rh1—C1	2.104 (4)	C12—H12B	0.9800
Rh1—C2	2.108 (4)	C13—C14	1.355 (6)
Rh1—C5	2.198 (4)	C13—H13	0.918 (19)
Rh1—C6	2.207 (4)	C14—C15	1.510 (6)
Rh1—Br1	2.5239 (5)	C14—H14	0.935 (18)
Rh2—C27	2.043 (3)	C15—C16	1.512 (7)
Rh2—C9	2.103 (4)	C15—H15A	0.9800
Rh2—C10	2.121 (4)	C15—H15B	0.9800
Rh2—C13	2.191 (4)	C16—H16A	0.9800
Rh2—C14	2.210 (4)	C16—H16B	0.9800
Rh2—Br2	2.5205 (5)	C18—C19	1.499 (6)
N1—C17	1.346 (5)	C18—H18A	0.9800
N1—C18	1.461 (5)	C18—H18B	0.9800
N1—C28	1.476 (5)	C19—C20	1.499 (6)
N4—C27	1.338 (5)	C19—H19A	0.9800
N4—C26	1.460 (5)	C19—H19B	0.9800
N4—C29	1.483 (5)	C20—H20A	0.9800
N2—C17	1.350 (5)	C20—H20B	0.9800
N2—C21	1.461 (5)	C21—C22	1.518 (5)
N2—C20	1.466 (5)	C21—H21A	0.9800
N3—C27	1.346 (5)	C21—H21B	0.9800
N3—C23	1.460 (5)	C22—C23	1.529 (6)
N3—C24	1.465 (5)	C22—H22A	0.9800
C1—C2	1.393 (7)	C22—H22B	0.9800
C1—C8	1.505 (7)	C23—H23A	0.9800
C1—H1	0.927 (19)	C23—H23B	0.9800
C2—C3	1.515 (6)	C24—C25	1.504 (6)
C2—H2	0.910 (19)	C24—H24A	0.9800
C3—C4	1.459 (8)	C24—H24B	0.9800
C3—H3A	0.9800	C25—C26	1.500 (6)
C3—H3B	0.9800	C25—H25A	0.9800
C4—C5	1.498 (7)	C25—H25B	0.9800
C4—H4A	0.9800	C26—H26A	0.9800
C4—H4B	0.9800	C26—H26B	0.9800
C5—C6	1.351 (7)	C28—C282	1.520 (6)
C5—H5	0.928 (19)	C28—C281	1.525 (6)
C6—C7	1.513 (7)	C28—H28	0.9900
C6—H6	0.930 (18)	C29—C291	1.511 (6)
C7—C8	1.463 (8)	C29—C292	1.519 (6)
C7—H7A	0.9800	C29—H29	0.9900
C7—H7B	0.9800	C281—H28A	0.9700
C8—H8A	0.9800	C281—H28B	0.9700
C8—H8B	0.9800	C281—H28C	0.9700
C9—C10	1.405 (6)	C282—H28D	0.9700
C9—C16	1.504 (6)	C282—H28E	0.9700
C9—H9	0.941 (18)	C282—H28F	0.9700
C10—C11	1.519 (6)	C291—H29A	0.9700
C10—H10	0.932 (19)	C291—H29B	0.9700
C11—C12	1.513 (8)	C291—H29C	0.9700
C11—H11A	0.9800	C292—H29D	0.9700
C11—H11B	0.9800	C292—H29E	0.9700
C12—C13	1.510 (7)	C292—H29F	0.9700
			
C17—Rh1—C1	90.89 (16)	C14—C13—H13	121 (3)
C17—Rh1—C2	91.66 (15)	C12—C13—H13	112 (3)
C1—Rh1—C2	38.61 (18)	Rh2—C13—H13	101 (3)
C17—Rh1—C5	161.56 (17)	C13—C14—C15	124.7 (4)
C1—Rh1—C5	94.95 (19)	C13—C14—Rh2	71.3 (2)
C2—Rh1—C5	82.20 (17)	C15—C14—Rh2	110.7 (3)
C17—Rh1—C6	162.59 (17)	C13—C14—H14	118 (2)
C1—Rh1—C6	80.86 (18)	C15—C14—H14	115 (2)
C2—Rh1—C6	91.34 (18)	Rh2—C14—H14	102 (2)
C5—Rh1—C6	35.71 (18)	C14—C15—C16	112.5 (3)
C17—Rh1—Br1	89.38 (10)	C14—C15—H15A	109.1
C1—Rh1—Br1	159.60 (13)	C16—C15—H15A	109.1
C2—Rh1—Br1	161.76 (14)	C14—C15—H15B	109.1
C5—Rh1—Br1	91.15 (12)	C16—C15—H15B	109.1
C6—Rh1—Br1	93.08 (13)	H15A—C15—H15B	107.8
C27—Rh2—C9	90.25 (15)	C9—C16—C15	114.0 (4)
C27—Rh2—C10	92.15 (16)	C9—C16—H16A	108.8
C9—Rh2—C10	38.84 (18)	C15—C16—H16A	108.8
C27—Rh2—C13	158.99 (15)	C9—C16—H16B	108.8
C9—Rh2—C13	97.05 (16)	C15—C16—H16B	108.8
C10—Rh2—C13	81.59 (17)	H16A—C16—H16B	107.7
C27—Rh2—C14	164.93 (15)	N1—C17—N2	117.8 (3)
C9—Rh2—C14	81.46 (16)	N1—C17—Rh1	122.6 (3)
C10—Rh2—C14	89.25 (17)	N2—C17—Rh1	119.6 (3)
C13—Rh2—C14	35.85 (15)	N1—C18—C19	110.0 (3)
C27—Rh2—Br2	89.27 (10)	N1—C18—H18A	109.7
C9—Rh2—Br2	158.18 (13)	C19—C18—H18A	109.7
C10—Rh2—Br2	162.95 (14)	N1—C18—H18B	109.7
C13—Rh2—Br2	91.07 (12)	C19—C18—H18B	109.7
C14—Rh2—Br2	93.78 (11)	H18A—C18—H18B	108.2
C17—N1—C18	122.7 (3)	C20—C19—C18	109.0 (4)
C17—N1—C28	120.1 (3)	C20—C19—H19A	109.9
C18—N1—C28	116.4 (3)	C18—C19—H19A	109.9
C27—N4—C26	122.3 (3)	C20—C19—H19B	109.9
C27—N4—C29	119.7 (3)	C18—C19—H19B	109.9
C26—N4—C29	117.4 (3)	H19A—C19—H19B	108.3
C17—N2—C21	119.2 (3)	N2—C20—C19	109.9 (3)
C17—N2—C20	124.6 (3)	N2—C20—H20A	109.7
C21—N2—C20	115.6 (3)	C19—C20—H20A	109.7
C27—N3—C23	119.8 (3)	N2—C20—H20B	109.7
C27—N3—C24	124.1 (3)	C19—C20—H20B	109.7
C23—N3—C24	115.3 (3)	H20A—C20—H20B	108.2
C2—C1—C8	125.8 (5)	N2—C21—C22	113.4 (3)
C2—C1—Rh1	70.9 (2)	N2—C21—H21A	108.9
C8—C1—Rh1	112.6 (3)	C22—C21—H21A	108.9
C2—C1—H1	114 (3)	N2—C21—H21B	108.9
C8—C1—H1	114 (3)	C22—C21—H21B	108.9
Rh1—C1—H1	110 (3)	H21A—C21—H21B	107.7
C1—C2—C3	123.7 (5)	C21—C22—C23	108.1 (3)
C1—C2—Rh1	70.5 (2)	C21—C22—H22A	110.1
C3—C2—Rh1	111.2 (3)	C23—C22—H22A	110.1
C1—C2—H2	122 (3)	C21—C22—H22B	110.1
C3—C2—H2	112 (3)	C23—C22—H22B	110.1
Rh1—C2—H2	105 (3)	H22A—C22—H22B	108.4
C4—C3—C2	117.2 (4)	N3—C23—C22	113.7 (3)
C4—C3—H3A	108.0	N3—C23—H23A	108.8
C2—C3—H3A	108.0	C22—C23—H23A	108.8
C4—C3—H3B	108.0	N3—C23—H23B	108.8
C2—C3—H3B	108.0	C22—C23—H23B	108.8
H3A—C3—H3B	107.2	H23A—C23—H23B	107.7
C3—C4—C5	115.6 (4)	N3—C24—C25	109.3 (3)
C3—C4—H4A	108.4	N3—C24—H24A	109.8
C5—C4—H4A	108.4	C25—C24—H24A	109.8
C3—C4—H4B	108.4	N3—C24—H24B	109.8
C5—C4—H4B	108.4	C25—C24—H24B	109.8
H4A—C4—H4B	107.4	H24A—C24—H24B	108.3
C6—C5—C4	126.5 (5)	C26—C25—C24	108.9 (3)
C6—C5—Rh1	72.5 (3)	C26—C25—H25A	109.9
C4—C5—Rh1	107.9 (3)	C24—C25—H25A	109.9
C6—C5—H5	119 (3)	C26—C25—H25B	109.9
C4—C5—H5	113 (3)	C24—C25—H25B	109.9
Rh1—C5—H5	99 (3)	H25A—C25—H25B	108.3
C5—C6—C7	125.3 (5)	N4—C26—C25	110.1 (3)
C5—C6—Rh1	71.8 (3)	N4—C26—H26A	109.6
C7—C6—Rh1	110.7 (3)	C25—C26—H26A	109.6
C5—C6—H6	114 (2)	N4—C26—H26B	109.6
C7—C6—H6	119 (2)	C25—C26—H26B	109.6
Rh1—C6—H6	101 (2)	H26A—C26—H26B	108.2
C8—C7—C6	115.8 (4)	N4—C27—N3	118.8 (3)
C8—C7—H7A	108.3	N4—C27—Rh2	123.1 (3)
C6—C7—H7A	108.3	N3—C27—Rh2	118.0 (2)
C8—C7—H7B	108.3	N1—C28—C282	110.4 (3)
C6—C7—H7B	108.3	N1—C28—C281	112.6 (3)
H7A—C7—H7B	107.4	C282—C28—C281	111.9 (3)
C7—C8—C1	115.7 (5)	N1—C28—H28	107.2
C7—C8—H8A	108.4	C282—C28—H28	107.2
C1—C8—H8A	108.4	C281—C28—H28	107.2
C7—C8—H8B	108.4	N4—C29—C291	113.0 (4)
C1—C8—H8B	108.4	N4—C29—C292	110.6 (3)
H8A—C8—H8B	107.4	C291—C29—C292	111.8 (4)
C10—C9—C16	126.3 (4)	N4—C29—H29	107.0
C10—C9—Rh2	71.3 (2)	C291—C29—H29	107.0
C16—C9—Rh2	109.8 (3)	C292—C29—H29	107.0
C10—C9—H9	114 (2)	C28—C281—H28A	109.5
C16—C9—H9	114 (2)	C28—C281—H28B	109.5
Rh2—C9—H9	111 (2)	H28A—C281—H28B	109.5
C9—C10—C11	124.2 (4)	C28—C281—H28C	109.5
C9—C10—Rh2	69.9 (2)	H28A—C281—H28C	109.5
C11—C10—Rh2	113.2 (3)	H28B—C281—H28C	109.5
C9—C10—H10	120 (3)	C28—C282—H28D	109.5
C11—C10—H10	112 (3)	C28—C282—H28E	109.5
Rh2—C10—H10	106 (3)	H28D—C282—H28E	109.5
C12—C11—C10	113.9 (4)	C28—C282—H28F	109.5
C12—C11—H11A	108.8	H28D—C282—H28F	109.5
C10—C11—H11A	108.8	H28E—C282—H28F	109.5
C12—C11—H11B	108.8	C29—C291—H29A	109.5
C10—C11—H11B	108.8	C29—C291—H29B	109.5
H11A—C11—H11B	107.7	H29A—C291—H29B	109.5
C13—C12—C11	113.6 (4)	C29—C291—H29C	109.5
C13—C12—H12A	108.8	H29A—C291—H29C	109.5
C11—C12—H12A	108.8	H29B—C291—H29C	109.5
C13—C12—H12B	108.8	C29—C292—H29D	109.5
C11—C12—H12B	108.8	C29—C292—H29E	109.5
H12A—C12—H12B	107.7	H29D—C292—H29E	109.5
C14—C13—C12	125.3 (4)	C29—C292—H29F	109.5
C14—C13—Rh2	72.8 (2)	H29D—C292—H29F	109.5
C12—C13—Rh2	108.6 (3)	H29E—C292—H29F	109.5

## References

[bb1] Blessing, R. H. (1995). *Acta Cryst.* A**51**, 33–38. 10.1107/s0108767394005726 7702794

[bb2] Díez-González, S., Marion, N. & Nolan, S. P. (2009). *Chem. Rev.* **109**, 3612–3676. 10.1021/cr900074m 19588961

[bb3] Evans, D., Osborn, J. A. & Wilkinson, G. J. (1968). *J. Chem. Soc. A*, pp. 3133–3142.

[bb4] Herrmann, W. A., Elison, M., Fischer, J., Köcher, C. & Artus, G. R. J. (1996). *Chem. Eur. J.* **2**, 772–780.

[bb5] Herrmann, W. A., Fischer, J., Köcher, C. & Artus, G. R. J. (1997). *J. Organomet. Chem.* **530**, 259–262.

[bb6] Huckaba, A. J., Hollis, T. K. & Reilly, S. W. (2013). *Organometallics*, **32**, 6248–6256.

[bb7] Mayr, M., Wurst, K., Ongania, K. & Buchmeiser, M. R. (2004). *Chem. Eur. J.* **10**, 1256–1266. 10.1002/chem.200305437 15007815

[bb8] Nonius (1998). *COLLECT* Nonius BV, Delft, The Netherlands.

[bb9] Otwinowski, Z. & Minor, W. (1997). *Methods in Enzymology*, Vol. 276, *Macromolecular Crystallography*, Part A, edited by C. W. Carter Jr & R. M. Sweet, pp. 307–326. New York: Academic Press.

[bb10] Reindl, S. A., Pöthig, A., Drees, M., Bechlars, B., Herdtweck, E., Herrmann, W. A. & Kühn, F. E. (2013). *Organometallics*, **32**, 4082–4091.

[bb11] Sheldrick, G. M. (2008). *Acta Cryst.* A**64**, 112–122. 10.1107/S0108767307043930 18156677

[bb12] Westrip, S. P. (2010). *J. Appl. Cryst.* **43**, 920–925.

